# Changes in Perceptions and Use of Mobile Technology and Health Communication in South Africa During the COVID-19 Lockdown: Cross-sectional Survey Study

**DOI:** 10.2196/25273

**Published:** 2021-05-17

**Authors:** Alex Emilio Fischer, Tanya Van Tonder, Siphamandla B Gumede, Samanta T Lalla-Edward

**Affiliations:** 1 Ezintsha Faculty of Health Sciences University of Witwatersrand Johannesburg South Africa; 2 Opinion Solutions Johannesburg South Africa

**Keywords:** coronavirus, SARS-CoV-2, COVID-19, technology, mHealth, app

## Abstract

**Background:**

In late March 2020, South Africa implemented a 5-stage COVID-19 Risk Adjusted Strategy, which included a lockdown that required all residents to remain home to prevent the spread of COVID-19. Due to this lockdown, individuals have been forced to find and use alternatives for accomplishing tasks including shopping, socializing, working, and finding information, and many have turned to the internet and their mobile devices.

**Objective:**

This study aimed to describe how South Africans consume and internalize information surrounding the COVID-19 outbreak in order to determine whether the COVID-19 lockdown and social isolation have influenced technology behavior, particularly in terms of health communication and information.

**Methods:**

From June 24 to August 24, 2020, people in South Africa were invited to complete a survey through the Upinion mobile app, an online data collection resource. The survey collected information on demographics, and technology use during the lockdown, and COVID-19 knowledge.

**Results:**

There were 405 participants, of which 296 (73.06%) were female. A total of 320 (79.01%) participants had a tertiary school education, 242 (59.75%) were single, and 173 (42.72%) had full-time employment. The lockdown forced 363 (89.63%) participants to use more technology, especially for work (n=140, 24.05%) and social media/communication (n=133, 22.85%). Security or privacy issues (n=46, 38.98%) and unfamiliarity with technology (n=32, 27.12%) were identified as the most common issues faced by the 127 (31.36%) participants who were unsure about using technology prior to the lockdown. Almost all participants (n=392, 96.79%) stated that they would continue using technology after the lockdown. Multimedia (n=215, 53.09%), mobile phone content (n=99, 24.44%), and health organizations and professionals (n=91, 22.47%) were the main sources of COVID-19 information. Most participants (n=282, 69.63%) felt that they had enough information. Two-thirds (n=275, 67.90%) of participants stated that they had used their mobile phones for health information before the lockdown, with web searches (n=109, 26.91%), social media (n=58, 14.32%), and government and institutional websites (n=52; 12.84%) serving as their main sources of information. Overall, the mean COVID-19 knowledge score was 8.8 (out of 10), and 335 (82.72%) had adequate knowledge (scored ≥8). Males were less likely to identify the correct transmission routes, and single participants were less likely to identify the signs and symptoms of the coronavirus. Tertiary school graduates were 4 times more likely to correctly identify the routes and 2 times more likely to identify how to stop the spread of the virus. People aged 43-56 years were 4 times more likely to identify how the coronavirus can be prevented, and participants ≥57 years were 2.6 times more likely to obtain a knowledge score of 10 when compared to those under 29 years of age.

**Conclusions:**

This study has shown that the COVID-19 lockdown has forced people to increase technology use, and people plan to continue using technology after the lockdown is lifted. Increased technology use was seen across a variety of fields; however, barriers including privacy, unfamiliarity, and data costs were identified. This population showed high COVID-19 knowledge, although the use of web searches and social media, instead of government and institutional websites, increases the potential for health misinformation to be spread.

## Introduction

On March 11, 2020, the World Health Organization declared the COVID-19 outbreak a pandemic [1]; thereafter, many countries followed China’s “lockdown” approach to reduce new cases. In March 2020, South Africa began a 21-day, level 5 lockdown as part of a 5-stage COVID-19 Risk Adjusted Strategy. During this period, only hospitals, clinics, grocery stores, and pharmacies remained open, and only essential personnel (eg, doctors, nurses, police) were permitted to leave their homes, although there were some controlled exceptions for medical care or essential supplies like food and medicine [2].

During the lockdown, cases and preventative measures have been well documented and investigated, both globally and in South Africa [3,4]. However, the behavioral effects of the lockdown are not as well known. With social distancing, individuals have been forced to find and use alternatives to accomplish tasks such as shopping, working, attending school, and staying informed, and many have turned to their mobile devices. In China there was a 30% increase in app use during their lockdown [5-7], while a global analysis of five popular social media platforms (Twitter, Instagram, YouTube, Reddit, and Gab) identified 8 million COVID-19–related posts and comments over the first 45 days of 2020 [8].

In a sense, this is all forced use of technology since people have limited alternatives to meet their needs, and to engage with this captive audience, many governments and institutions have introduced mobile health (mHealth) interventions to disseminate information during the pandemic [9-12]. Specifically, the South African government has implemented a website that provides information on COVID-19, the Risk Adjusted Strategy, preventative measures, news and updates, and links to other resources [13]. These additional resources include a WhatsApp support line, an emergency telehealth hotline, social media message campaigns, and updates from the South African Government and National Department of Health websites [3,13,14].

With all of this electronic communication resulting from COVID-19, researchers have taken the opportunity to investigate how it has influenced digital health, and a variety of studies have already been conducted. Some studies have harnessed big data to predict outbreak hotspots with algorithm-based web mining [8,15-17], while others have looked at how individuals share and consume COVID-19–related content [18]. A study from India showed that more than half of participants (n=58, 56.3%) had adequate information regarding COVID-19; however, their primary source of information was from multimedia (radio, TV, newspaper) (n=57, 55.3%), and only 22 (21.4%) relied on the internet as their main source for information [19].

Despite high mobile penetration in low- and middle-income countries [20,21], there are still individuals who have not embraced technology for various reasons, including security and privacy concerns, data costs, and an inability to understand modern electronics [22]. With the limitations set by the lockdown, increased exposure to technology may have altered some people’s perceptions and use of technology. This study aimed to describe how South Africans consume and internalize information surrounding the COVID-19 outbreak to assess whether the COVID-19 lockdown and social isolation has influenced technology behavior, particularly for health communication and information.

## Methods

### Study Design

This South African cross-sectional study was conducted electronically, administered through the Upinion mobile app, an online data collection resource. Participants were included if they were an existing or new Upinion user with current access to surveys on the app, ≥18 years of age, and able to provide online consent. Individuals were excluded if they were not able to access the Upinion app, were younger than 18 years, or refused to participate.

### Data Collection

From June 24 to August 24, 2020, existing and new Upinion users were invited to complete a survey through Upinion notifications and advertisements on social media platforms, respectively. Once an individual agreed to participate in the current study, they were able to provide informed consent through the app and then register for the survey group [23]. The participant was then given access to the survey, which was completed through their mobile phone. During the survey, all answers were recorded electronically in the backend of the app.

A mobile app was used to collect data as this was deemed the easiest way to gather responses, while obeying the lockdown restrictions and ensuring the safety of both participants and data collectors. This method of online distribution of a survey and accompanying electronic consent has been used with increasing frequency, particularly during the COVID-19 pandemic for studies with similar methodologies [19,24-27].

### The Upinion App

**Figure 1:**
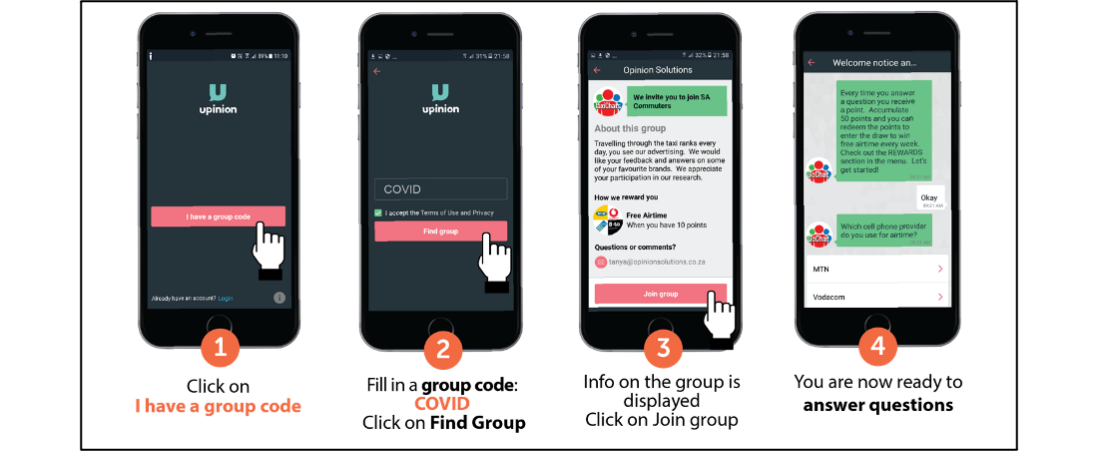
The Upinion app.

The Upinion messaging and data collection app was developed in 2014 by Upinion, a people-centric research technology company based in the Netherlands, and its use in Southern African Development Community countries is licensed to Opinion Solutions. The app was developed as a way to collect feedback from affected communities in any response effort in order to provide better and more efficient support. It serves as an outlet for those affected by crisis to share their unique problems, needs and solutions, so that nongovernmental organizations have a grass-roots understanding of the situation on the ground, allowing for tailored interventions. This has been used by nonprofit organizations like Oxfam to identify the needs of refugee communities [23], and research institutes like the Wits Reproductive Health and HIV Institute to administer health-related surveys directly via participants’ mobile phones [28]. Upinion does not collect personal data, but rather personal data is collected through survey questions and the participant shares this voluntarily. Upinion encrypts all mobile phone numbers and IP addresses in compliance with General Data Protection Regulation and is also ISO/IEC (International Organization for Standardization/International Electrotechnical Commission) 27001 certified. Screenshots of the Upinion app are presented in Figure 1.

### Survey Development

This survey was adapted from the survey *Demographic Data and Structure Knowledge Questionnaire Regarding Prevention of COVID-19*, used in a similar study from India [19]. The original questionnaire consists of two sections—the first comprising 8 questions to explore demographic information and the second 10 questions that focused on COVID-19 knowledge. In our survey (Multimedia Appendix 1), we have modified the sections on demographic information and COVID-19 knowledge to reflect the South African context, and a third section was added to explore participants’ technology use during the COVID-19 outbreak.

### Data Analysis

Upinion has a built-in dashboard to monitor responses in real time; however, the final data set was exported to Excel (Microsoft Corp) for cleaning and coding, then exported to Stata V.15 (StataCorp) for analysis. Demographic information, technology use, and COVID-19 knowledge questions were all described as frequency and percentages. A mean knowledge score (with standard deviation) was also calculated across all 10 knowledge questions, with a score below 6 considered inadequate knowledge, 6-8 considered moderately adequate knowledge, and a score above 8 considered adequate knowledge [19].

The Pearson chi-square test was used to assess trends of association between outcome variables (COVID-19 knowledge and technology use) and demographic characteristics. Logistic regression models (bivariate [not included in this paper] and multivariable models) were constructed for the outcome variables to control for confounders and identify independent predictors. These predictors were reported as crude (not included in this paper) and adjusted odds ratios (aOR), with 95% CI and *P* values (<.05 was considered significant).

### Ethical Consideration and Approval

Ethics approval was obtained from the University of the Witwatersrand Human Research Ethics Committee (nonmedical) (reference number 200512). Survey respondents did not receive any compensation for participation.

## Results

### Demographics

Participants’ demographic data are presented in Table 1. Of the 405 participants, 84 (20.74%) were 28 years or younger, 165 (40.74%) were between the ages of 29 and 42 years, 110 (27.16%) were between the ages of 43 and 56 years, and 46 (11.36%) were 57 years or older. There were 296 (73.06%) females, 320 (79.01%) participants had completed tertiary school education, and 242 (59.75%) were single. A total of 173 (42.72%) participants had full-time employment, 74 (18.27%) were casually employed, 29 (7.16%) were students, and 129 (32.85%) were unemployed.

**Table 1:** Demographic characteristics.

Characteristic		Participants (N=405), n (%)^a^
**Age (years)**		
	18-28	84 (20.74)
	29-42	165 (40.74)
	43-56	110 (27.16)
	≥57	46 (11.36)
**Sex**		
	Female	296 (73.09)
	Male	109 (26.91)
**Education**		
	Primary school or less	1 (0.25)
	Secondary school	84 (20.74)
	Tertiary school (any)	320 (79.01)
**Marital status**		
	Married	163 (40.25)
	Single	242 (59.75)
**Employment status**		
	Casually employed	74 (18.27)
	Full-time employment	173 (42.72)
	Student	29 (7.16)
	Unemployed	129 (31.85)

^a^Percentages may not add up to 100.00% due to rounding.

### Technology Use

A total of 363 (89.63%) participants stated that the lockdown had forced them to use more technology, and the greatest increases in use were for work (n=140, 24.05%), social media/communication (n=133, 22.85%), shopping (n=78, 13.4%), and news and information (n=70, 12.03%). Nearly one-third (n=127, 31.36%) of participants stated that they were unsure about using technology before the lockdown, with security and privacy issues (n=46, 38.98%) and unfamiliarity with technology (n=32, 27.12%) identified as the most common concerns. More than half (n=209, 51.60%) the participants had positive feelings about the increased forced technology use, while almost all (n=392, 96.79%) participants stated that they would continue using technology after the lockdown. When asked about information regarding COVID-19, 282 (69.63%) felt that they had enough information and knowledge, with multimedia (n=215, 53.09%), mobile phone content (n=99, 24.44%), and health organizations and professionals (n=91, 22.47%) as their main source of COVID-19 information. Two-thirds (n=275, 67.90%) of participants stated that they had used their mobile phones for health information before the COVID-19 outbreak, with web searches (n=109, 26.91%), social media posts (n=58,14.32%), government and institutional websites (n=52, 12.84%), and mobile apps (n=58, 14.32%) serving as their main sources of health information (Table 2).

**Table 2:** Technology use.

Technology questions				Participants (N=405), n (%)^a^
**Has the lockdown forced you to use more technology?**				
	Yes			363 (89.63)
	No			42 (10.37)
	**If yes, what do you use technology for?**			
		Job searching	33 (5.67)	
		Social media/communication	133 (22.85)	
		Education	58 (9.97)	
		Shopping	78 (13.40)	
		Entertainment	48 (8.25)	
		Work	140 (24.05)	
		News and information	70 (12.03)	
		Banking	16 (2.75)	
		Religion	6 (1.03)	
**Were you unsure about using technology/online methods before?**				
	Yes			127 (31.36)
	No			278 (68.64)
	**If yes, what made you feel uncomfortable?**			
		Security/privacy issues	46 (38.98)	
		Unfamiliar with technology	32 (27.12)	
		Lack of personal connection/accountability	16 (13.56)	
		Cost of data and devices	10 (8.47)	
		Reliability issues	14 (11.86)	
**How do you feel about the increased forced use of technology?**				
	Positive feelings			209 (51.60)
	Neutral/mixed feelings			129 (31.85)
	Negative feelings			67 (16.54)
**Will you continue to use technology after the lockdown?**				
	Yes			392 (96.79)
	No			13 (3.21)
**Do you have enough information/knowledge regarding COVID-19?**				
	Yes			282 (69.63)
	No			123 (30.37)
**What is your main source of information on COVID-19?**				
	Health organizations and professionals			91 (22.47)
	Mobile phone content			99 (24.44)
	Multimedia (radio, television, newspaper)			215 (53.09)
**Have you used your mobile phone for health information before the COVID-19 outbreak?**				
	Yes			275 (67.90)
	No			130 (32.10)
	**If yes, what was your main source of health information?**			
		Email	1 (0.25)	
		Government/institutional websites	52 (12.84)	
		Messaging platforms (WhatsApp, SMS)	17 (4.20)	
		Mobile apps	38 (9.38)	
		Social media posts	58 (14.32)	
		Web searches (Google)	109 (26.91)	

^a^Percentages may not add up to 100.00% due to rounding.

Logistic regression analysis identified relationships between demographics and 4 technology use variables (Multimedia Appendix 2). When asked if the lockdown had forced participants to use more technology, participants with a tertiary school education were 2.5 times more likely to increase their technology use than those with a primary or secondary school education (aOR 2.580; 95% CI 1.212-5.489, *P*=.01), and full-time employees were also less likely to increase their technology use compared to those casually employed (aOR 0.275; 95% CI 0.078-0.966, *P*=.04).

Regarding the main source of COVID-19 information, multimedia, health organizations and professionals, and mobile phone content all had demographic associations. Tertiary school graduates were less likely to use multimedia as their main source of COVID-19 information compared to those with primary or secondary school education (aOR 0.536; 95% CI 0.319-0.900, *P*=.02). Multimedia was almost 2 times more likely to be the main source of information in respondents aged 29-42 years, when compared to those younger than 29 years (aOR 1.862; 95% CI 1.062-3.378, *P*=.04). Single participants were less likely to use health organizations and professionals (aOR 0.537; 95% CI 0.318-0.906, *P*=.02) as their main source of COVID-19 information. Mobile phone content was also associated with age, with the 57-70–year-old group being least likely (aOR 0.339; 95% CI 0.128-0.896, *P*=.03) to use their mobile as the main source of health information compared to those younger than 29 years of age.

The associations seen among participants who responded that they had enough information or knowledge about COVID-19 included age, being male, being single, and having a tertiary education.

The 57-70–year-old group were approximately 6 times (aOR 5.661; 95% CI 1.894-16.925, *P*=.002) more likely to have adequate information compared to those younger than 29 years of age. Males were almost twice as likely (aOR 1.892; 95% CI 1.094-3.272, *P*=.02) than females to have enough COVID-19 information as were those having a tertiary school education (aOR 1.885; 95% CI 1.111-3.198, *P*=.02) over those with a secondary education or lower, while single participants were less likely (aOR 0.509; 95% CI 0.297-0.873, *P*=.01) to have adequate information.

The oldest age group and students were the least likely to use their phone for health information prior to the pandemic (older adults: aOR 0.184; 95% CI 0.075-0.449, *P*<.001; students: aOR 0.277; 95% CI 0.103-0.740, *P*=.01).

### COVID-19 Knowledge

When asked about COVID-19, 358 (88.40%) participants correctly identified it as a contagious respiratory virus, and 392 (96.79%) correctly stated that it was transmitted through respiratory droplets. Over three-quarters (n=319, 78.77%) of participants correctly chose all the ways that the virus could be spread; the rest thought it was only spread by coughing or sneezing (n=52, 18.84%), by touching objects that have COVID-19 droplets on them (n=17, 4.2%), or through close contact with an infected individual (n=16, 3.95%). All of the common COVID-19 symptoms (cough, sore throat, fever, and shortness of breath) were correctly identified by 379 (93.58%) participants; the same percentage correctly identified all encouraged prevention techniques (avoid touching one’s face, avoid contact with sick people, and wash hands thoroughly). When asked about handwashing duration, 20 seconds was correctly selected by the majority (n=340, 83.95%). For the question on how to stop the spread of COVID-19, 368 (90.86%) correctly chose social distancing, self-isolation, and regular handwashing as their response, and when asked how to stop the chance of spreading the virus, 383 (94.57%) correctly chose coughing and sneezing into their elbow, social distancing and self-isolation, and regular handwashing as their response. Most participants (n=308, 76.05%) correctly stated that they would call the emergency hotline or WhatsApp support line if they thought they had COVID-19 symptoms, although 79 (19.51%) incorrectly stated that they would rush to the nearest hospital for testing. Lastly, practicing social distancing, self-isolation, and washing one’s hands thoroughly were all correctly identified by 369 (91.11%) participants as the key to prevent the spread of COVID-19 (Table 3).

**Table 3:** Structured COVID-19 questionnaire.

COVID-19 questions			Participants (N=405), n (%)^a^
**What is the novel coronavirus (COVID-19)?**			
	It is a bioweapon	11 (2.72)	
	It is a sexually transmitted infection	4 (0.99)	
	It is a very contagious respiratory virus	358 (88.40)	
	It is just another term for the common cold	22 (5.43)	
	It is transmitted through respiratory droplets	10 (2.47)	
**What are the transmission routes of COVID-19?**			
	It is transmitted by eating Chinese food	4 (0.99)	
	It is transmitted through direct blood contact	6 (1.48)	
	It is transmitted through respiratory droplets	392 (96.79)	
	It is transmitted through sexual intercourse	3 (0.74)	
**How can COVID-19 be spread?**			
	By touching objects that have COVID-19 respiratory droplets	17 (4.20)	
	Through close contact with an infected individual	16 (3.95)	
	Through coughing or sneezing	52 (12.84)	
	All of the above	319 (78.77)	
	(Blank)	1 (0.25)	
**What are the signs and symptoms of COVID-19?**			
	Cough and sore throat	11 (2.72)	
	Fever	15 (3.70)	
	Shortness of breath	12 (2.96)	
	All of the above	367 (90.62)	
**The coronavirus can be prevented by**			
	Avoid touching your face	8 (1.98)	
	Avoiding contact with sick people	7 (1.73)	
	Wash your hands thoroughly	11 (2.72)	
	All of the above	379 (93.58)	
**Wash your hands with soap or sanitizer for at least**			
	5 seconds	5 (1.23)	
	10 seconds	17 (4.20)	
	20 seconds	340 (83.95)	
	1 minute	43 (10.62)	
**To stop the spread of the coronavirus, you should**			
	Practice social distancing	17 (4.20)	
	Practice social distancing and wash your hands thoroughly	1 (0.25)	
	Self-isolate	16 (3.95)	
	Self-isolate and practice social distancing	1 (0.25)	
	Wash your hands thoroughly	2 (0.49)	
	All of the above	368 (90.86)	
**How can you stop the chances of spreading the coronavirus?**			
	Cough or sneeze into a tissue or your elbow	4 (0.99)	
	Self-isolate and practice social distancing	13 (3.21)	
	Wash your hands thoroughly	5 (1.23)	
	All of the above	383 (94.57)	
**What will you do if you suspect that you have symptoms of COVID-19?**			
	Call the emergency hotline or WhatsApp support line	308 (76.05)	
	Go to the pharmacy to get medication	9 (2.22)	
	Rush to the nearest hospital for testing	79 (19.51)	
	Stay in close physical contact with friends/family for support	8 (1.98)	
	(Blank)	1 (0.25)	
**What is the key to prevent the spread of COVID-19?**			
	Practice social distancing	10 (2.47)	
	Self-isolate	18 (4.44)	
	Wash your hands thoroughly	7 (1.73)	
	All of the above	369 (91.11)	
**Total knowledge score**			
	Inadequate (score ≤5)	19 (4.69)	
	Moderately adequate (score=6,7)	51 (12.59)	
	Adequate (score ≥8)	335 (82.72)	

^a^Percentages may not add up to 100.00% due to rounding.

Overall, the mean knowledge score was 8.8 (SD 1.53). There were only 19 (4.69%) participants with inadequate knowledge, 51 (12.59%) with moderately adequate knowledge, and 335 (82.72%) with adequate knowledge (Table 3).

Logistic regression analysis identified relationships between demographics and 4 COVID-19 knowledge variables. Males were less likely to identify the correct transmission routes of COVID-19 (aOR 0.216; 95% CI 0.063-0.744, *P*=.02) than females, while those with a tertiary education were 4 times more likely to correctly identify the routes (aOR 4.414; 95% CI 1.308-14.900, *P*=.02) than those with only primary or secondary education. Tertiary school graduates were also 2 times more likely to identify how to stop the spread of the virus (aOR 2.215; 95% CI 1.041-4.714, *P*=.04), compared to participants with only primary or secondary education. Single participants were less likely to identify the signs and symptoms of COVID-19 (aOR 0.182; 95% CI 0.052-0.631, *P*=.01) than married participants. The 43-56 years age category was 4 times more likely to identify how COVID-19 can be prevented (aOR 3.987; 95% CI 1.011-15.718, *P*=.048) compared to those under 29 years of age (Multimedia Appendix 3).

Lastly, association analyses conducted separately between demographics and the outcome variables (COVID-19 knowledge scores and technology use) only identified a significant relationship in participants ≥57 years being 2.6 times more likely to obtain a knowledge score of 10 (aOR 2.60; 95% CI 1.1-6.0, *P*=.03) when compared to participants 28 years and under.

## Discussion

### Principal Findings

This study is the first to describe how South Africans interact with technology and consume health information during the current COVID-19 outbreak. Our findings were in line with a similar study from India [19]. Multimedia was the main source of COVID-19 information for both countries (India: n=57, 55.4% vs South Africa: n=215, 53.09%), followed by the internet in India (n=22, 21.4%) and mobile phone content in South Africa (n=99, 24.44%). Despite more people in India stating that they had adequate COVID-19 information (India: n=98, 95.1% vs South Africa: n=282, 69.63%), the South African mean knowledge score of 8.8 was slightly higher than that of India (8.01). The South African study also showed that the lockdown has forced the majority of participants to increase their technology use and these findings are in line with similar increases in technology use from around the world [5-7,29-31]. Participants with a tertiary school education were more likely to increase their technology use than those with less education, who were less likely to use multimedia as their main source of COVID-19 information. This is in line with a study from sub-Saharan Africa, which showed that the positive effects of mobile phone use is diminished by poor primary education [32]. However, in addition, these findings may be explained by socioeconomic factors associated with more education, as college graduates earn higher wages and are better equipped to cope with economic shocks [33]. Full-time employees were less likely to increase their technology use compared to those who were causally employed, although this may be due to a higher baseline of technology use for full-time employees due to the growing demands of the knowledge economy [34].

The rise in South African technology use has also been validated by the nation’s data usage, which increased by more than one-third over the first few days of the lockdown [35]. This increase in technology use led the government to quickly digitize education through a combination of free electronic readers and zero-rated educational apps and websites. This has allowed schools to move to an online curriculum, which has facilitated the return to studies via home-based schooling for many students, by mid-March 2020 [29]. Similarly, apps and websites are also being used by the National Department of Health to relay COVID-19 information to the public [3,13,14] ; however, there are many other online sources for COVID-19 information.

Government or institutional websites [3,4,13,14] publish evidence-based information and fact check their findings; however, more participants stated that their main mobile source of health information was web searches or social media posts. Unfortunately, web searches and social media posts are not regulated, and the sharing of misinformation has created an infodemic surrounding COVID-19 [8,25]. This misinformation includes false news articles, conspiracy theories surrounding the virus creation, ineffective home remedies for treatment, and downplaying of the need for prevention control, such as social distancing and mask use. The propagation of this misinformation can actually present a health risk and may undermine the countermeasures implemented by governments and credible institutions [8,36]. Despite a high overall knowledge score, misinformation may have played a role in this study, as two questions (*How COVID-19 can be spread?* and *What will you do if you suspect that you have symptoms of COVID-19?)* scored below adequate. These questions may identify knowledge gaps where increased outreach is needed to educate the population, especially for the second question, where 79 (19.51%) participants stated that they would rush to the nearest hospital for testing instead of calling the emergency hotline or WhatsApp support line for further instructions. There are a number of documented ways to engage users on mobile platforms, and the government can use them to dispel misinformation by guiding people to accurate information sources. Social media outreach, with dialogue loops, is a particularly effective way to engage with individuals, and this type of social media outreach can be tailored with specific messages that target specific subpopulations [37,38].

Misinformation may have disproportionately affected participants under the age of 29 years, especially when compared to those above 57 years. The older group was less likely to use their mobile as the main source of health information, yet they were 6 times more likely to have enough COVID-19 information, and 2.6 times more likely to obtain a knowledge score of 10. In South Africa, youth under 30 years are almost 20% more likely to use their phone to access the internet than their parents, which would expose the younger age group to more online misinformation than the oldest age group [39]. Single participants were less likely to use health organizations and professionals as their source of COVID-19 information, and not using a trusted source may have also led to misinformation, as they were less likely to have enough COVID-19 information and correctly identify COVID-19 signs and symptoms. Having enough COVID-19 information may not be a true indicator of knowledge though since males were twice as likely to say they had enough COVID-19 information but were less likely to identify the correct routes of COVID-19 transmission.

This study has also reiterated some known barriers to mobile use in South Africa, such as security and privacy issues, unfamiliarity with technology, and data costs. Due to an increase in data usage, some local networks have temporarily lowered data costs [35], but long-term affordable data plans are required to ensure equitable mobile usage for the duration of this lockdown and in the future [40]. Security and privacy issues have been well documented in South Africa, especially for mHealth platforms [22,41,42]. However, previous studies have shown that personal identification number (PIN)–protected mobile platforms for delivering sensitive health information are feasible and acceptable in South Africa [42,43]. Furthermore, a Japanese study that investigated online consumption suggests that the process of making online purchases for the first time during the lockdown has facilitated people becoming familiar with technology, thus alleviating some perceived barriers [44]. This information provides context to the 392 (96.8%) participants who stated they will continue to use technology after the pandemic. However, follow-up studies must be conducted to quantify this.

### Limitations

A selection bias may be present due to the device and data requirements needed to access this survey, which was conducted online via a convenience sample. As this survey was adapted from a pre-existing survey, it was not validated or pilot tested in South Africa before this study. Furthermore, participants were asked to self-report their technology use, and no measurements were taken to validate these statements.

### Conclusion

This study has shown that the COVID-19 lockdown has forced many people to increase technology use, and almost all participants will continue to use technology post lockdown. Increased technology use was seen across a variety of fields; however, well-known barriers were cited, including privacy and security concerns, unfamiliarity with technology, and data costs. This population showed high COVID-19 knowledge, but the use of web searches and social media posts, instead of government and institutional websites, provides the potential for health misinformation about COVID-19 to be spread. This was particularly evident in some subdemographic groups, including participants under 29 years, single participants, participants without tertiary education, and males. These groups should be targeted with further education and preventative measures.

## Appendix

Multimedia Appendix 1 Technology use during the COVID-19 lockdown survey.

Multimedia Appendix 2 Logistic regressions of technology use.

Multimedia Appendix 3 Logistic regressions of COVID-19 knowledge.

## Abbreviations

aOR: adjusted odds ratio
IEC: International Electrotechnical Commission
ISO: International Organization for Standardization
mHealth: mobile health
