# Supplementary material for: Changes in Perceptions and Use of Mobile Technology and Health Communication in South Africa During the COVID-19 Lockdown: Cross-sectional Survey Study
Source: JMIR Form Res. 2021 May 17;5(5):e25273. doi: 10.2196/25273 (PMC8130817; doi:10.2196/25273)
Supplement: Multimedia Appendix 1 [file formative_v5i5e25273_app1.docx]

**Multimedia Appendix 1. Technology use during the COVID-19 lockdown survey.**

| Section 1. Demographic and mHealth familiarity | |
| --- | --- |
| How old are you? | a) 14-28  b) 29-42  c) 43-56  d) 57-70 |
| What gender are you? | a) Male  b) Female  c) Other |
| What is your educational status? | a) Primary school or less  b) Secondary school  c) Tertiary school (any) |
| What is your marital status? | a) Single  b) Married |
| What is your employment status? | a) Student  b) Unemployed  c) Casually employed  d) Full-time employment |
| Section 2. Technology use | |
| Has the lock down forced you to use more technology? | a) Yes  b) No |
| If yes, what do you use technology for? | ____________ |
| Where you unsure about using technology/ online methods before? | a) Yes  b) No |
| If yes, what made you feel uncomfortable? | ____________ |
| How do you feel about the increased forced use of technology? | ____________ |
| Will you continue to use technology after the lock down? | a) Yes  b) No |
| Do you have enough information/knowledge regarding COVID-19? | a) Yes  b) No |
| What is your main source of information for COVID-19? | a) Multimedia (Radio, television, newspaper)  b) Health organizations and professionals  c) Mobile phone content |
| Have you used your mobile phone for health information before the COVID-19 outbreak? | a) Yes  b) No |
| If yes, what was your main source of health information? | a) Mobile apps  b) Social media posts  c) Messaging platforms (WhatsApp, SMS  d) Web searches (ex. Google)  e) Government/institutional websites (ex. NDOH, WHO, CDC)  f) email |
| Section 3. Structured COVID-19 questionnaire | |
| What is Novel Coronavirus (COVID-19)? | a) It is a bioweapon  b) It is a very contagious respiratory virus  c) It is a sexually transmitted infection  d) It is just another term for the common cold  e) It is transmitted through respiratory droplets |
| What are transmission routes of COVID-19 | a) It is transmitted through respiratory droplets  b)It is transmitted through direct blood contact  c) It is transmitted through sexual intercourse  d) It is transmitted by eating Chinese food |
| How COVID-19 can be spread? | a) Through coughing or sneezing  b) By touching objects that have COVID respiratory droplets  c) Through close contact with an infected individual  d) All of the above |
| What are the signs and symptoms of coronavirus? | a) Fever  b) Cough and sore throat  c) Shortness of breath  d) All of the above |
| Corona virus can be prevented by | a) Avoiding contact with sick people  b) avoid touching your face  c) Wash your hands thoroughly  d) All of the above |
| Wash your hands with soap or sanitizer for at least | a) 5 seconds  b) 10 seconds  c) 20 seconds  d) 1 minute |
| To stop spread corona virus you should | a) Self isolate  b) Practice social distancing  c) Wash your hands thoroughly  d) All of the above |
| How can you stop the chance of spreading corona virus? | a) Self isolate and practice social distancing  b) Cough or sneeze into a tissue or your elbow  c) Wash your hands thoroughly  d) All of the above |
| What you will do when suspected that you have symptoms of coronavirus? | a) Rush to nearest hospital for testing  b) Call emergency hotline or WhatsApp support line  c) Stay in close physical contact with friends/family for support  d) Go to the pharmacy to get medication |
| Important key to prevent from spreading of COVID-19 is? | a) Self isolate  b) Practice social distancing  c) Wash your hands thoroughly  d) All of the above |
