# Supplementary material for: Changes in Perceptions and Use of Mobile Technology and Health Communication in South Africa During the COVID-19 Lockdown: Cross-sectional Survey Study
Source: JMIR Form Res. 2021 May 17;5(5):e25273. doi: 10.2196/25273 (PMC8130817; doi:10.2196/25273)
Supplement: Multimedia Appendix 2 [file formative_v5i5e25273_app2.docx]

**Table 3. Logistic regression of Technology use**

| **Variable** | **Has the lock down forced you to use more technology? (y/n)** | | | **Multimedia was the main source of SARS-C0V-2 information. (y/n)** | | | **Health organizations/ professionals was the main source of SARS-COV-2 information. (y/n)** | | | **Mobile phone content was the main source of SARS-C0V-2 information. (y/n)** | | | **Will you continue to use technology after the lock down? (y/n)** | | | **Do you have enough information/knowledge regarding SARS-CoV-2? (y/n)** | | | **Have you used your mobile phone for health information before the SARS-CoV-2 outbreak? (y/n)** | | |
| --- | --- | --- | --- | --- | --- | --- | --- | --- | --- | --- | --- | --- | --- | --- | --- | --- | --- | --- | --- | --- | --- |
|  | **AOR** | **95% CI** | ***p*** | **AOR** | **95% CI** | ***p*** | **AOR** | **95% CI** | ***p*** | **AOR** | **95% CI** | ***p*** | **AOR** | **95% CI** | ***p*** | **AOR** | **95% CI** | ***p*** | **AOR** | **95% CI** | ***p*** |
| **Age** |  |  |  |  |  |  |  |  |  |  |  |  |  |  |  |  |  |  |  |  |  |
| 18-28 | Ref | - | - | Ref | - | - | Ref | - | - | Ref | - | - | Ref | - | - | Ref | - | - | Ref | - | - |
| 29-42 | 0.538 | 0.163-1.777 | .31 | 1.862 | 1.026-3.378 | **.04** | 1.039 | 0.463-2.331 | .93 | 0.497 | 0.263-0.942 | **.03** | 1.070 | 0.197-5.819 | .94 | 1.846 | 1.003-3.397 | **.049** | 0.605 | 0.305-1.202 | .15 |
| 43-56 | 0.539 | 0.147-1.970 | .35 | 1.477 | 0.750-2.909 | .26 | 1.523 | 0.646-3.592 | .34 | 0.454 | 0.213-0.971 | **.04** | 1.887 | 0.247-14.409 | .54 | 3.722 | 1.749-7.921 | **.001** | 0.295 | 0.136-0.638 | **.002** |
| 57-70 | 0.312 | 0.076-1.276 | .11 | 2.227 | 0.985-5.033 | .05 | 1.194 | 0.437-3.265 | .73 | 0.339 | 0.128-0.896 | **.03** | 1.614 | 0.122-21.316 | .72 | 5.661 | 1.894-16.925 | **.002** | 0.184 | 0.075-0.449 | **<.001** |
| **Gender** |  |  |  |  |  |  |  |  |  |  |  |  |  |  |  |  |  |  |  |  |  |
| Female | Ref | - | - | Ref | - | - | Ref | - | - | Ref | - | - | Ref | - | - | Ref | - | - | Ref | - | - |
| Male | 1.012 | 0.467-2.191 | .98 | 0.972 | 0.617-1.533 | .90 | 0.806 | 0.453-1.433 | .46 | 1.234 | 0.736-2.069 | .43 | 2.349 | 0.498-11.089 | .28 | 1.892 | 1.094-3.272 | **.02** | 1.444 | 0.864-2.414 | .16 |
| **Relationship status** |  |  |  |  |  |  |  |  |  |  |  |  |  |  |  |  |  |  |  |  |  |
| Married | Ref | - | - | Ref | - | - | Ref | - | - | Ref | - | - | Ref | - | - | Ref | - | - | Ref | - | - |
| Single | 1.188 | 0.587-2.403 | .63 | 1.398 | 0.891-2.193 | .15 | 0.537 | 0.318-0.906 | **.02** | 1.192 | 0.691-2.057 | .53 | 0.914 | 0.257-3.249 | .89 | 0.509 | 0.297-0.873 | **.01** | 0.615 | 0.373-1.015 | .06 |
| **Education** |  |  |  |  |  |  |  |  |  |  |  |  |  |  |  |  |  |  |  |  |  |
| Primary/secondary | Ref | - | - | 1 | - | - | 1 | - | - | 1 | - | - | 1 | - | - | 1 | - | - | 1 | - | - |
| Tertiary | 2.580 | 1.212-5.489 | **.01** | 0.536 | 0.319-0.900 | **.02** | 1.381 | 0.703-2.713 | .35 | 1.788 | 0.959-3.335 | .07 | 0.288 | 0.036-2.322 | .24 | 1.885 | 1.111-3.198 | **.02** | 1.337 | 0.777-2.299 | .29 |
| **Employment status** |  |  |  |  |  |  |  |  |  |  |  |  |  |  |  |  |  |  |  |  |  |
| Casually employed | Ref | - | - | Ref | - | - | Ref | - | - | Ref | - | - | Ref | - | - | Ref | - | - | Ref | - | - |
| Full-time employment | 0.275 | 0.078-0.966 | **.04** | 1.085 | 0.614-1.917 | .78 | 1.142 | 0.589-2.214 | .70 | 0.760 | 0.390-1.480 | .42 | 0.275 | 0.032-2.332 | .24 | 1.271 | 0.661-2.446 | .47 | 1.086 | 0.571-2.067 | .80 |
| Student | 0.807 | 0.074-8.799 | .86 | 1.671 | 0.658-4.243 | .28 | 0.368 | 0.075-1.810 | .22 | 0.894 | 0.334-2.393 | .82 | 0.207 | 0.015-2.737 | .23 | 1.066 | 0.413-2.753 | .90 | 0.277 | 0.103-0.740 | **.01** |
| Unemployed | 0.338 | 0.094-1.223 | .098 | 1.240 | 0.692-2.224 | .47 | 0.681 | 0.330-1.403 | .30 | 1.034 | 0.535-1.997 | .92 | 0.841 | 0.075-9.496 | .89 | 1.025 | 0.534-1.966 | .94 | 0.592 | 0.311-1.127 | .11 |

Abbreviation: y/n=yes/no, AOR=Adjusted Odds Ratios,95% CI=95% Confidence Interval, *P*=*P* value
