# Supplementary material for: Changes in Perceptions and Use of Mobile Technology and Health Communication in South Africa During the COVID-19 Lockdown: Cross-sectional Survey Study
Source: JMIR Form Res. 2021 May 17;5(5):e25273. doi: 10.2196/25273 (PMC8130817; doi:10.2196/25273)
Supplement: Multimedia Appendix 3 [file formative_v5i5e25273_app3.docx]

**Table 5. Logistic regression of COVID-19 knowledge**

| **Variable** | **Correctly answered ‘What are transmission routes of COVID-19?’ (y/n)** | | | **Correctly answered ‘How the coronavirus can be spread?’ (y/n)** | | | **Correctly answered ‘What are coronavirus signs and symptoms?’ (y/n)** | | | **Correctly answered ‘Corona virus can be prevented by?’ (y/n)** | | | **Correctly answered ‘To stop spread corona virus you should’(y/n)** | | | **Correctly answered ‘How can you stop the chance of spreading corona virus?’ (y/n)** | | |
| --- | --- | --- | --- | --- | --- | --- | --- | --- | --- | --- | --- | --- | --- | --- | --- | --- | --- | --- |
|  | **AOR** | **95% CI** | ***P*** | **AOR** | **95% CI** | ***P*** | **AOR** | **95% CI** | ***P*** | **AOR** | **95% CI** | ***P*** | **AOR** | **95% CI** | ***P*** | **AOR** | **95% CI** | ***P*** |
| **Age** |  |  |  |  |  |  |  |  |  |  |  |  |  |  |  |  |  |  |
| 14-28 | Ref | - | - | Ref | - | - | Ref | - | - | Ref | - | - | Ref | - | - | Ref | - | - |
| 29-42 | 2.253 | 0.545-9.342 | .26 | 0.887 | 0.457-1.719 | .72 | 1.185 | 0.524-2.680 | .68 | 2.729 | 0.955-7.802 | .06 | 0.840 | 0.309-2.282 | .73 | 0.759 | 0.228-2.526 | .65 |
| 43-56 | 1.790 | 0.271-11.805 | .55 | 1.661 | 0.722-3.821 | .23 | 2.844 | 0.809-9.993 | .10 | 3.987 | 1.011-15.718 | **.048** | 1.182 | 0.356-3.928 | .79 | 2.238 | 0.354-14.142 | .39 |
| 57-70 | 1 | - | - | 1.640 | 0.586-4.589 | .35 | 1 |  | - | 3.008 | 0.549-16.477 | .20 | 1.199 | 0.263-5.471 | .82 | 0.914 | 0.141-5.919 | .93 |
| **Gender** |  |  |  |  |  |  |  |  |  |  |  |  |  |  |  |  |  |  |
| Female | Ref | - | - | Ref | - | - | Ref | - | - | Ref | - | - | Ref | - | - | Ref | - | - |
| Male | 0.216 | 0.063-0.744 | **.02** | 0.958 | 0.554-1.659 | .88 | 0.836 | 0.393-1.780 | .64 | 0.957 | 0.390-2.347 | .92 | 0.829 | 0.385-1.782 | .63 | 0.444 | 0.177-1.117 | .09 |
| **Relationship status** |  |  |  |  |  |  |  |  |  |  |  |  |  |  |  |  |  |  |
| Married | Ref | - | - | Ref | - | - | Ref | - | - | Ref | - | - | Ref | - | - | Ref | - | - |
| Single | 0.253 | 0.028-2.333 | .23 | 0.742 | 0.417-1.318 | .31 | 0.182 | 0.052-0.631 | **.01** | 1.057 | 0.652-3.998 | .91 | 1.099 | 0.504-2.395 | .81 | 0.633 | 0.199-2.007 | .44 |
| **Education** |  |  |  |  |  |  |  |  |  |  |  |  |  |  |  |  |  |  |
| Primary/secondary | 1 | - | - | 1 | - | - | 1 | - | - | 1 | - | - | 1 | - | - | Ref | - | - |
| Tertiary | 4.414 | 1.308-14.900 | **.02** | 0.852 | 0.468-1.553 | .60 | 1.113 | 0.509-2.434 | .79 | 1.615 | 0.652-3.998 | .30 | 2.215 | 1.041-4.714 | **.04** | 1.608 | 0.594-4.355 | .35 |
| **Employment status** |  |  |  |  |  |  |  |  |  |  |  |  |  |  |  |  |  |  |
| Casually employed | Ref | - | - | Ref | - | - | Ref | - | - | Ref | - | - | Ref | - | - | Ref |  | - |
| Full-time employment | 1.665 | 0.232-11.943 | .61 | 1.808 | 0.905-3.611 | .09 | 1.562 | 0.566-4.310 | .39 | 0.650 | 0.184-2.287 | .50 | 0.614 | 0.210-1.789 | .37 | 1.293 | 0.454-3.680 | .63 |
| Student | 1.404 | 0.188-10.504 | .74 | 1.137 | 0.411-3.143 | .81 | 1.710 | 0.457-6.403 | .43 | 0.607 | 0.132-2.796 | .52 | 0.477 | 0.108-2.101 | .33 | 0.494 | 0.132-1.845 | .29 |
| Unemployed | 0.708 | 0.157-3.192 | .65 | 0.994 | 0.512-1.927 | .99 | 1.068 | 0.427-2.675 | .89 | 0.850 | 0.243-2.967 | .80 | 0.789 | 0.261-2.387 | .67 | 1 | - | - |

Abbreviation: y/n=yes/no, AOR=Adjusted Odds Ratios,95% CI=95% Confidence Interval, *P*=*P* value
